# Supplementary material for: Removal of false positives in metagenomics-based taxonomy profiling via targeting Type IIB restriction sites
Source: Nat Commun. 2023 Sep 1;14:5321. doi: 10.1038/s41467-023-41099-8 (PMC10474111; doi:10.1038/s41467-023-41099-8)
Supplement: Supplementary file 1 — Supplementary Information [file 41467_2023_41099_MOESM1_ESM.pdf]

Removal of false positives in metagenomic-based taxonomy profiling via targeting Type IIB restriction sites

Supplementary Information

Supplemental Methods

Supplemental Figures

Figure S1. Distribution of theoretically existent and unique 2b tags for all the 48,475 species in the combined database of GTDB and Ensemble Fungi.

Figure S2. Sample-specific unique 2b database can largely expand the number of unique 2b tags compared to pre-constructed unique 2b database.

Figure S3. Comparing MAP2B with other profilers using simulation data generated by shared genome sources of different profilers.

Figure S4. Benchmarking MAP2B using simulation data generated by genome sources independent of GTDB or with a high mutation rate.

Figure S5. Assessing MAP2B's microbial profiles in disease discrimination (validation cohort) and prediction of metabolomic profiles (using mNODE).

Figure S6. Comparison of the algorithm implemented in MAP2B and conventional metagenomic profiling tools.

Figure S7. Definition of the terminology used in the species identification procedures of MAP2B.

Figure S8. Top-10 feature combinations in each machine learning classifier when selecting the false positive recognition model.

Figure S9. Performance evaluations of the machine learning model on false positive recognition based on 50 times five-fold cross-validation.

Reference

Supplemental Methods

Feature engineering and selection for the false positive recognition model

We sought to resolve the false-positive identification issue by leveraging a classifier that relies on meaningful biological features (**Fig. S7**). Consider the *in silico* digestion of reference genomes and a given WMS dataset. For species-i in the integrated database of GTDB[1] and Ensembl Fungi[2], we denote its total number of 2b tags generated by *in silico* digestion of its genome as H_i, representing the number of its theoretically existent 2b tags. Among the H_i tags, there are E_i unique 2b tags, which are single-copy within species-i's genome and are unique to species-i w.r.t all other species in the database.

Given an input WMS dataset with total R reads, we *in silico* extract 2b tags, map them to the species-specific 2b tags, and denote the number of tags unique to species-i as Q_i (sequenced unique 2b tags). Among the Q_i tags unique to species-i, there are U_i nonredundant (or distinct) sequenced unique 2b tags.

The genome coverage of species-i can be calculated as $C_i = U_i/E_i$. Usually, we have $C_i < 1$ for WMS data of complex microbial communities. But we can infer the actual number of sequenced unique 2b tags of species-i, denoted as $\tilde{Q}_i$, by its genome coverage correction, i.e., $\tilde{Q}_i = Q_i/C_i = (Q_iE_i)/U_i$.

Now we propose seven features to help us distinguish false positives from true positives. Those seven features can be classified into three distinct categories:

(1) Related to the genome coverage:

$$\text{Feature 1} = C_i = \frac{U_i}{E_i}, (\text{genome coverage}) \qquad (7)$$

(2) Related to the taxonomic and sequence abundance:

$$\text{Feature 2} = N_i = \frac{Q_i}{U_i}, (\text{taxonomic counts}) \qquad (8)$$

$$\text{Feature 3} = \frac{Q_i}{H_i}, \qquad (9)$$

$$\text{Feature 4} = \frac{Q_i}{R}, \qquad (10)$$

$$\text{Feature 5} = R_i = \frac{\tilde{Q}_i}{R}, (\text{sequence counts}) \qquad (11)$$

(3) Related to both genome coverage and abundances:

$$\text{Feature 6} = \sqrt{\frac{U_i}{E_i} * \frac{\tilde{Q}_i}{R}}, \qquad (12)$$

$$\text{Feature 7} = G_i = \sqrt{Q_i * U_i}, (\text{G} - \text{score}) \qquad (13)$$

We then employed min-max scaling and log transformation separately for any combination of the above seven features before passing to six classifiers in the scikit-learn 1.1.0 (Logistic Regression, Support-Vector Machines, naive Bayes, K-neighbors, AdaBoost, as well as

Gradient Boosting Classifier) to select the best feature combination and the best classifier. Specifically, for each feature combination using each normalization and classifier, we performed five-fold cross-validation five times using the CAMI2 simulation datasets. As a result, we found that the Random Forest classifier using log transformed features 1,2,5, and 7 (i.e., genome coverage C_i, taxonomic count N_i, sequence count R_i, and G-score G_i) has the best performance (**Fig. S8**).

Generating a GTDB version ground truth for CAMI 2 simulation datasets

CAMI 2 simulation datasets were synthetized using novel assembled microbial genomes as source genomes with only Refseq annotations[3]. By merging CAMI 2's source genomes with GTDB and annotating them with GTDB-TK, we were able to generate a GTDB version ground truth for MAP2B as well as improve the machine learning model's training accuracy (please see our GitHub repository for ground truth, see Manuscript/Figures/FigureS9/CAMI_50_abundance_change_in_ground_truth_100W.zip, "GT_Abd").

Validation of the false positive and true positive classifier

As the most important component of MAP2B, the classification of false positives and true positives largely determines the accuracy of the final profiling result. To best utilize the four key features (genome coverage, sequence count, taxonomic count, and G-score) for distinguishing true positives from false positives, we trained a Random Forest model based on these features using all the simulated metagenomes from CAMI2. Specifically, we randomly selected 80% of the samples in each of the three CAMI2 datasets (marine, plant-associated, and strain madness datasets) to train a Random Forest model with default parameters, then tested its performance using the remaining 20% of samples. We repeated the whole process of five-fold cross-validation 50 times by randomly assigning samples in either train or test folds. When evaluating the performance of the model, the low-abundance species in the ground truth were gradually filtered out according to varying abundance thresholds from 10^{-6} to 10^{-4}. This is because the sensitivity for species identification can be limited due to low sequencing depth (e.g., ~2GB/sample for the strain madness dataset). Some state-of-the-art metagenomic profilers[4, 5] actually set the default abundance threshold as 10^{-4}. Note that the minimum abundance in the CAMI2 datasets is 2×10^{-6}. Therefore, using threshold 10^{-6} is equivalent to not setting any threshold.

We separately evaluated the performance of the Random Forest classification model on each of the three simulation datasets. For the marine dataset, when using threshold 10^{-6} (or equivalently, without setting any threshold), we achieved Accuracy~0.988, AUROC~0.999, AUPRC~0.999, Precision~0.991, Recall~0.990, and F1 score~0.990, respectively (**Fig. S9a-f**). We then found that filtering out the species in the ground truth with abundance less than 10^{-5} will further increase the performance of the model. For example, in the marine dataset, the average of Accuracy, AUROC, AUPRC, Precision, Recall, and F1 increased to 0.989, 0.999, 0.999, 0.991, 0.990, and 0.991, respectively. Furthermore, filtering out species with

relative abundance less than 10^{-4} in the ground truth can maximize the performance of the model for the marine datasets (Accuracy, AUROC, AUPRC, Precision, Recall, and F1 are 0.992, 1, 1, 0.991, 0.995, and 0.993, respectively). A similar trend was observed for the plant-associated (green lines and dots in **Fig. S9**) and strain madness datasets (yellow lines and dots in **Fig. S9**). The average Accuracy, AUROC, AUPRC, Precision, Recall, and F1 for the three datasets (when using 10^{-4} as the threshold) are 0.993, 1, 0.997, 0.958, 0.975, and 0.966, respectively. Finally, the well-trained classifier with the best performance among 50 repeats will be used in MAP2B.

Usage of metagenomic profilers

For WMS data, we compared MAP2B with five state-of-the-arts metagenomic profilers: MetaPhlAn4[6], mOTUs3[7], Bracken[5], Kraken2[4], and KrakenUniq[8]. The detailed procedures are listed below.

(1) MetaPhlAn4 (v4.0.1) is a marker-gene alignment approach that relies on a precomputed databases containing clade-specific marker genes. Query reads are aligned via bowtie2 to the marker genes for microbial identification and abundance estimation. The database version used is mpa_vJan21_CHOCOPhlAnSGB_202103. The following MetaPhlAn4 command was used.

    "*metaphlan input_1.fastq.gz,input_2.fastq.gz --input_type fastq --bowtie2out output.bz2 --tax_lev s --nproc 32 -o output.txt*"

(2) mOTUs3 (v3.0.3, database version v3.0.3) is a marker-based method that compiles a large variety of phylogenetic marker genes from multiple biomes. Query reads are aligned using bwa mem and further processed to generate an abundance profile. The following mOTUs3 command was used.

    "*motus profile -f input_1.fastq.gz -r input_2.fastq.gz -n sample_name -u -p -k mOTU -o output.txt -t 32*"

(3) Kraken2 (v2.1.1) is a k-mers based taxonomic classification method. It searches for 35bp k-mers from the query sequence in a precomputed database that matches k-mers to the lowest common ancestor (LCA) taxon of all genomes that contain that taxon. The database was constructed using complete bacterial, archaeal, human and viral genomes from NCBI RefSeq (2020 Dec). A filtering abundance threshold of 0.01 (default) was selected. The following Kraken2 command was used.

    "*kraken2 --threads 32 --fastq-input --gzip-compressed --paired input_1.fastq.gz input_2.fastq.gz –output output.reads --report output.report*"

(4) Bracken (v2.5) utilizes the read classification output from standard Kraken for a Bayesian re-estimation of taxonomic abundances, which significantly improves the false-positive issue of standard Kraken and implicitly normalizes for genome length. The kraken-filter was used to filter raw classifications at the 0.01 threshold. The below Bracken command was used.

    "*est_abundance.py -i output_kraken2.report -k db -o output*"

(5) KrakenUniq (v0.5.8) is a metagenomics classifier that combines the fast k-mer-based classification of Kraken with an efficient algorithm for assessing the coverage of unique k-mers found in each species in a dataset. The database was constructed using complete

bacterial, archaeal and viral genomes from NCBI RefSeq (2022 Jan). A filtering abundance threshold of 0.01 (same with kraken2) was selected. The KrakenUniq command below was used.

"*krakenuniq --db db --threads 32 --report-file output.report --gzip-compressed input_1.fastq.gz input_2.fastq.gz --fastq-input*"

(6) As for MAP2B (v1), the default MAP2B command of "*perl MAP2B.pl -t 2 -l data.list -d MAP2B_DB_GTDB -o output*" was used.

Supplemental Figures

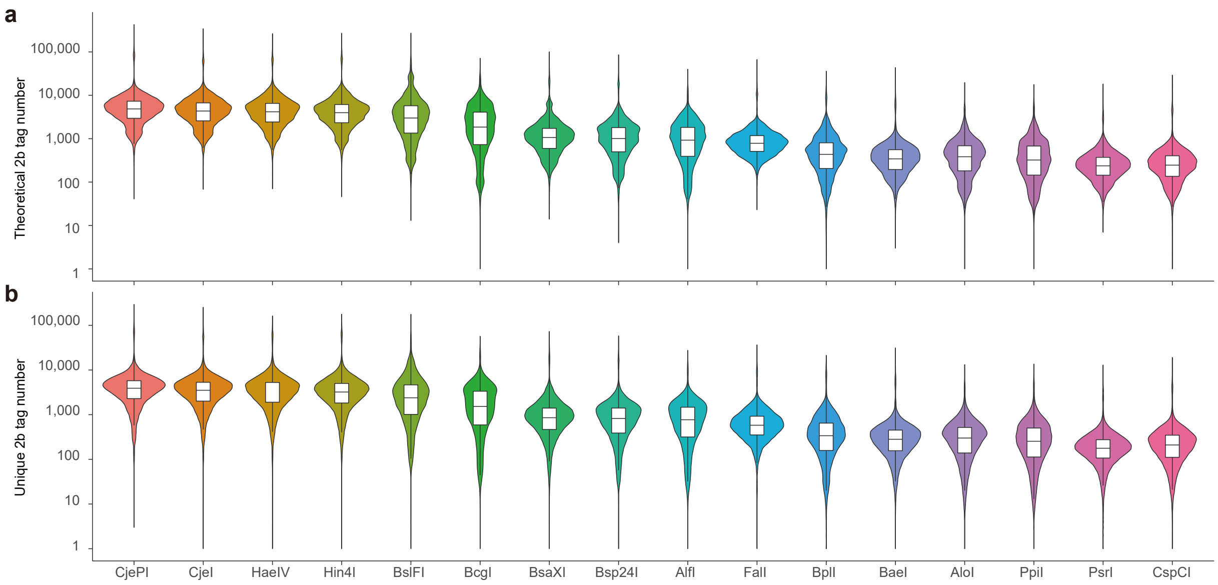

Figure S1. Distribution of theoretically existent and unique 2b tags for all the 48,475 species in the combined database of GTDB and Ensemble Fungi. (**a**) The theoretically existent 2b tags (H_i) were generated by *in silico* digestion (using 16 different Type IIB enzymes) for 48,475 species' 258,406 microbial genomes downloaded from GTDB and Ensembl Fungi. For species-i, its theoretically existent 2b tags in the integrated database of GTDB and Ensembl Fungi is denoted as H_i. (**b**) We then selected those 2b tags that are not duplicated/overlapped between any two species and named them as unique 2b tags. For species-i, its unique 2b tags in the integrated database of GTDB and Ensembl Fungi is denoted as E_i. The Type IIB restriction enzymes in X-axis are sorted by the median H_i (or E_i) in descending order. In this paper, we used *CjePI* as the Type IIB enzyme for *in silico* digestion since it has the highest median H_i and E_i. Using multiple IIB enzymes has limited improvement in the accuracy of species identification and abundance estimation.

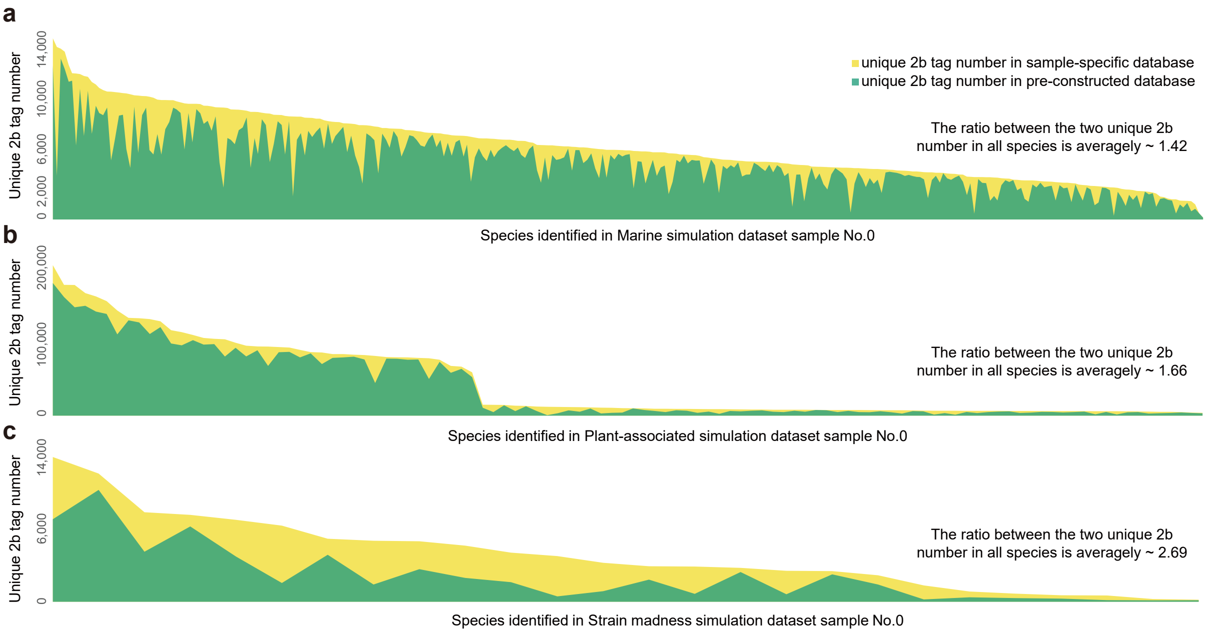

Figure S2. Sample-specific unique 2b database can largely expand the number of unique 2b tags compared to pre-constructed unique 2b database. To demonstrate the advantage of using a sample-specific unique 2b tag database for the second-round reads alignment, we consider the three CAMI2 simulation datasets of (**a**) marine, (**b**) plant-associated, and (**c**) strain madness as examples. We compare the number of unique 2b tags for all identified species between the preconstructed unique 2b tag database and sample-specific database and found that the average fold change for all the identified species is 1.42, 1.66, and 2.69 in the three datasets, respectively. The former contains unique 2b tags generated by comparing theoretically existent 2b tags among 48,475 species. By contrast, the latter usually contains twice unique 2b tags selected from a few hundreds of species.

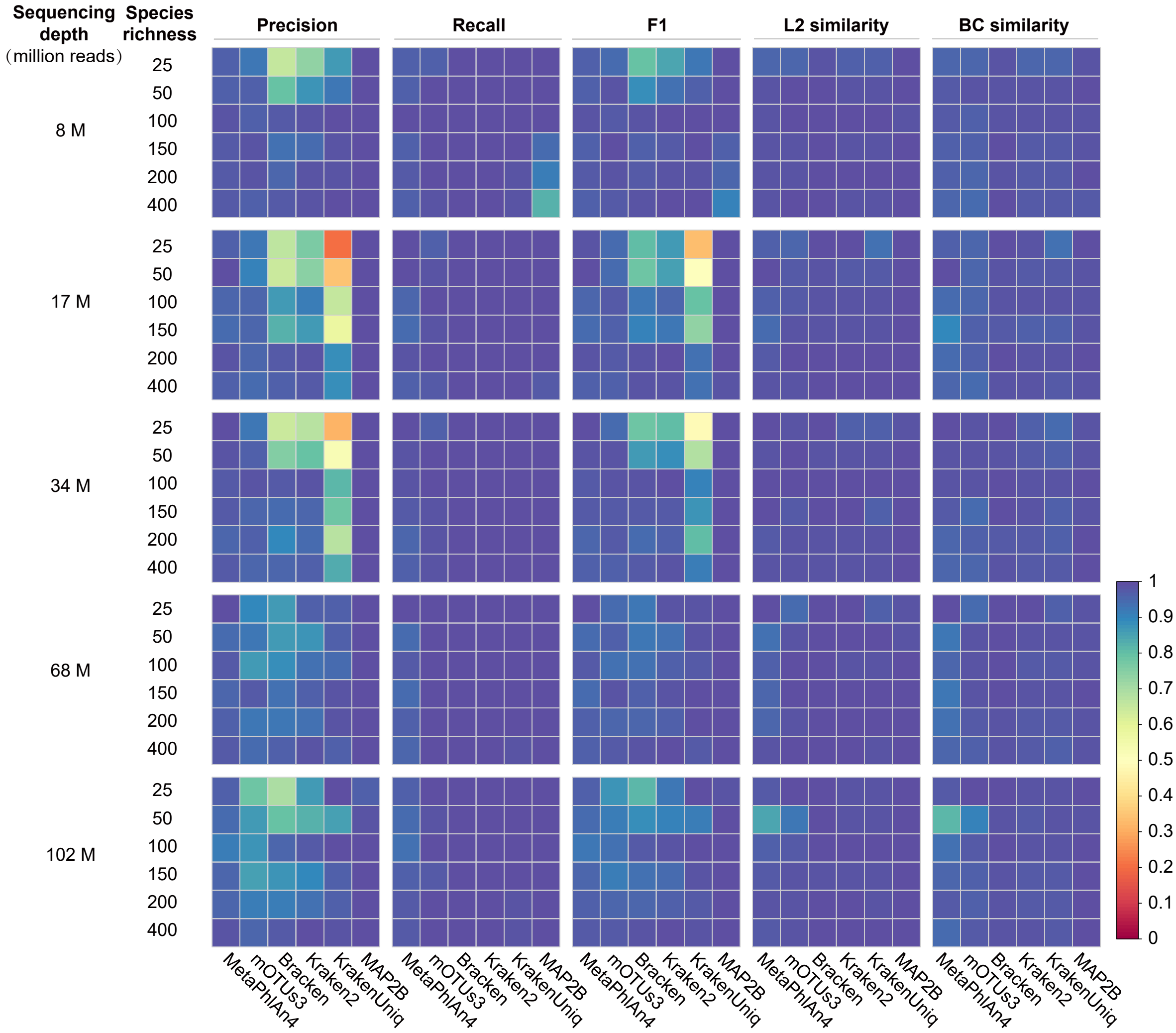

Figure S3. Comparing MAP2B with other profilers using simulation data generated by shared genome sources of different profilers. To minimize the influence of different reference databases on the evaluation, we selected the shared microbial genomes between different metagenomic profilers (e.g., mOTU2, MetaPhlaAn, and Kraken) as genomes to simulate WMS data. From left to right, the profiling results generated by different metagenomic profilers were compared with ground truth and illustrated by the Precision, Recall, F1 score, L2 similarity, and BC similarity. From top to bottom, the simulated sequencing depth increases from 8M to 102M, and the species richness increases from 25 to 400 under each sequencing depth. Since selecting the intersection of different metagenomic profilers' reference genomes dramatically decreased the number of source genomes for simulation, we slightly adjusted the species number and sequencing depth compared to Fig. 3.

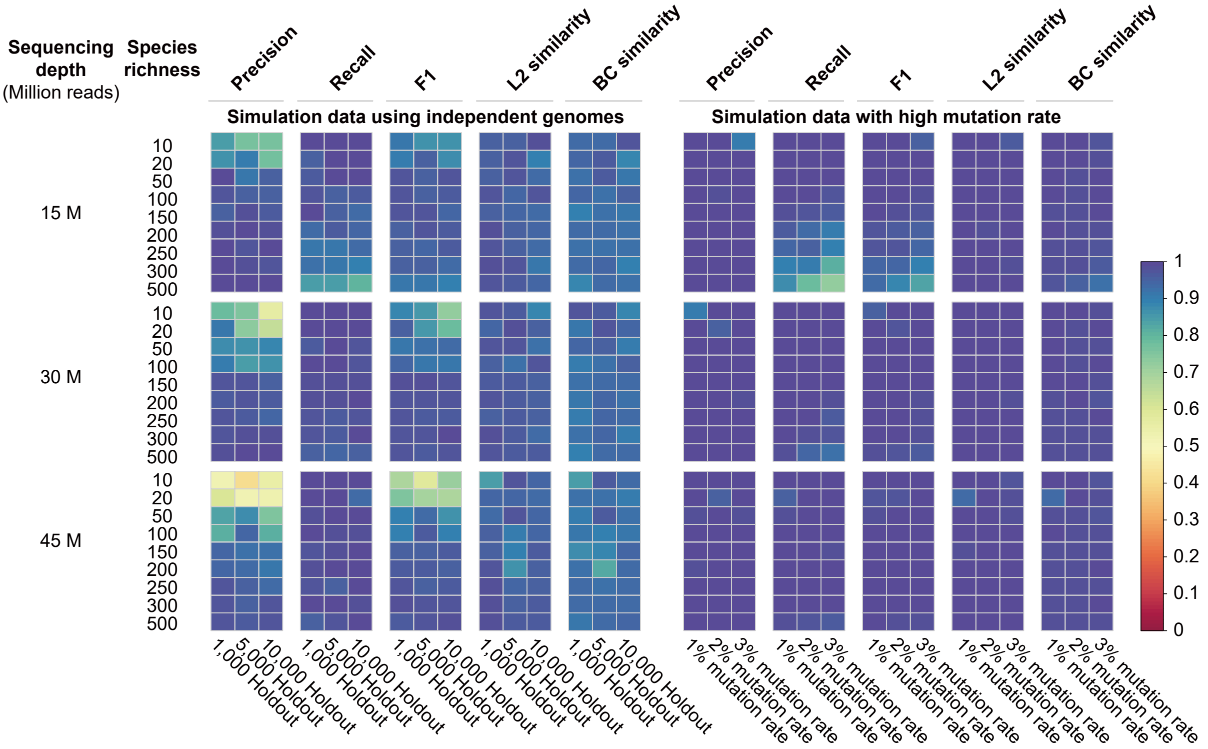

Figure S4. Benchmarking MAP2B using simulation data generated by genome sources independent of GTDB or with high a mutation rate. To overcome the challenge of accurately estimating species abundance in the absence of microbial genomes in the reference database, we implemented a systematic partitioning approach for the GTDB database. We utilized held-out genomes to simulate whole metagenome sequencing (WMS) data and systematically varied the mutation rate in the simulated data. The resulting profiles were compared to ground truth using Precision, Recall, F1 score, L2 similarity, and BC similarity metrics. We tested different numbers of holdout genomes and mutation rates, and the results were illustrated from left to right. In this evaluation, we also increased the simulated sequencing depth from 15M to 45M and increased species richness from 10 to 500 for each sequencing depth.

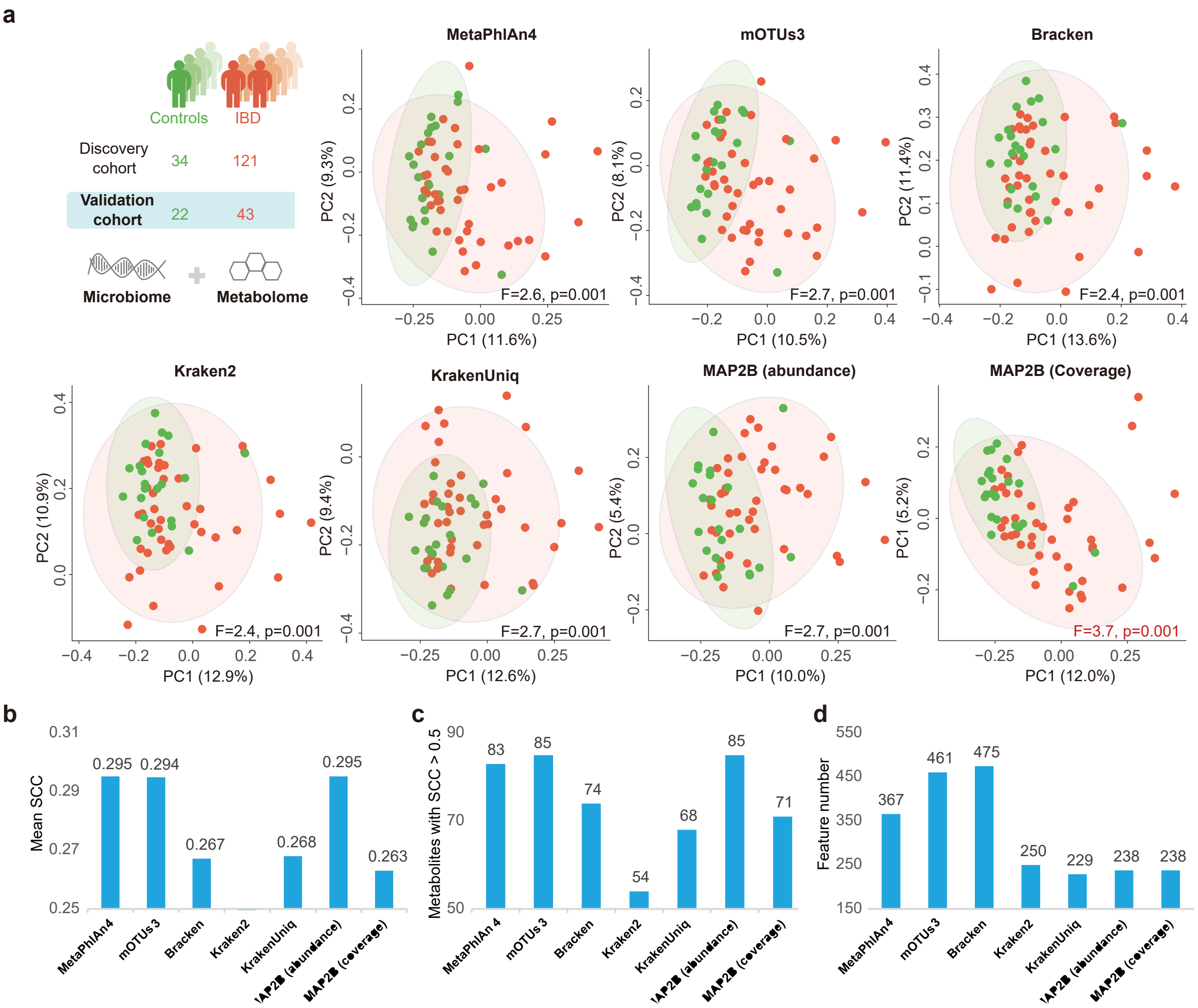

Figure S5. Assessing MAP2B's microbial profiles in disease discrimination (validation cohort) and prediction of metabolomic profiles. (**a**) PCoA plots for the validation cohort (n=65) based on the taxonomic profiles generated by different profilers. The ellipses with 95% CI are drawn to illustrate the difference between IBD (red dots) and non-IBD (green dots) in PCoA. F values of the PERMANOVA are also marked on the bottom of each plot to quantify the difference in disease status. (**b**) Comparison of prediction results by using different taxonomic profiling via mean SCC of the metabolite between its true values and predicted values across all individuals in the validation cohort. (**c**) Comparison of the number of metabolites with SCCs larger than 0.5 among different taxonomic profiling results. (**d**) Comparison of the number of taxonomic features used by different metagenomic profilers in the prediction for metabolomic profiles. The prediction results in (**b**) - (**c**) were generated by the mNODE which is in line with MiMeNet (**Fig. 5b-c**).

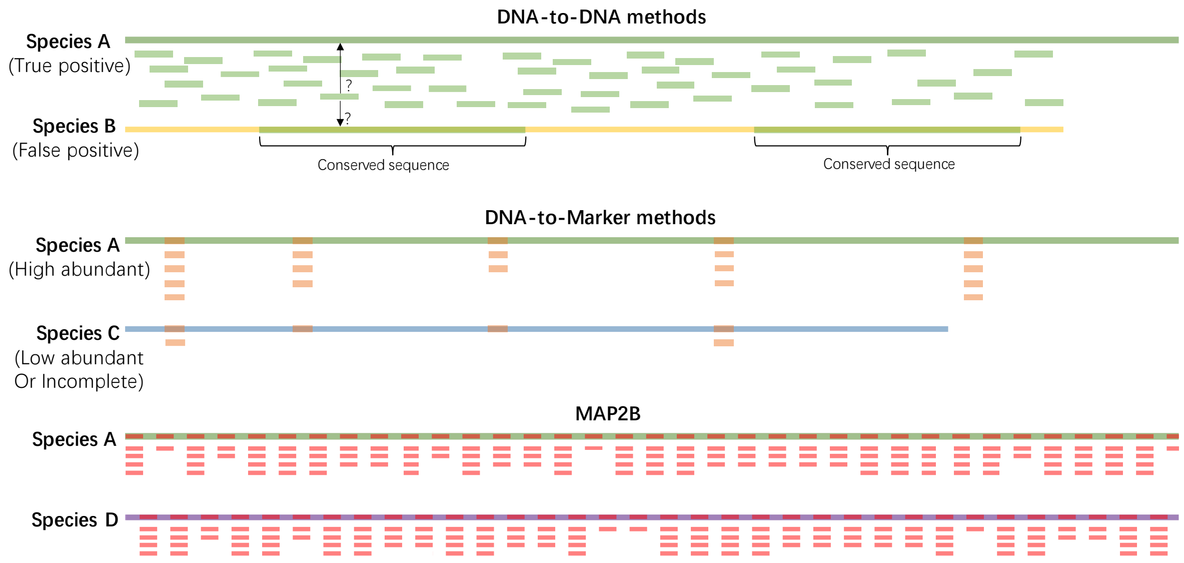

Figure S6. Comparison of the algorithm implemented in MAP2B and conventional metagenomic profiling tools. Using the whole genome as a reference, DNA-to-DNA methods (such as Bracken, Kraken, CLARK, Centrifuge, and PathSeq) may be confused by multi-alignments in conserved sequences, leading to a high rate of false positives. Although DNA-to-Marker methods (such as MetaPhlAn and mOTUs) can naturally avoid this issue, they may be limited by the availability of universal markers, such as missing markers from incomplete microbial genomes during database construction, high marker similarity among conspecific taxa during database construction (and sequencing), and undetectable markers in low abundant taxa during sequencing. MAP2B is not relying on the whole genome or universal marker genes as references. Using species-specific 2b tags can also avoid the multi-alignment issue while providing ample small markers for species identification.

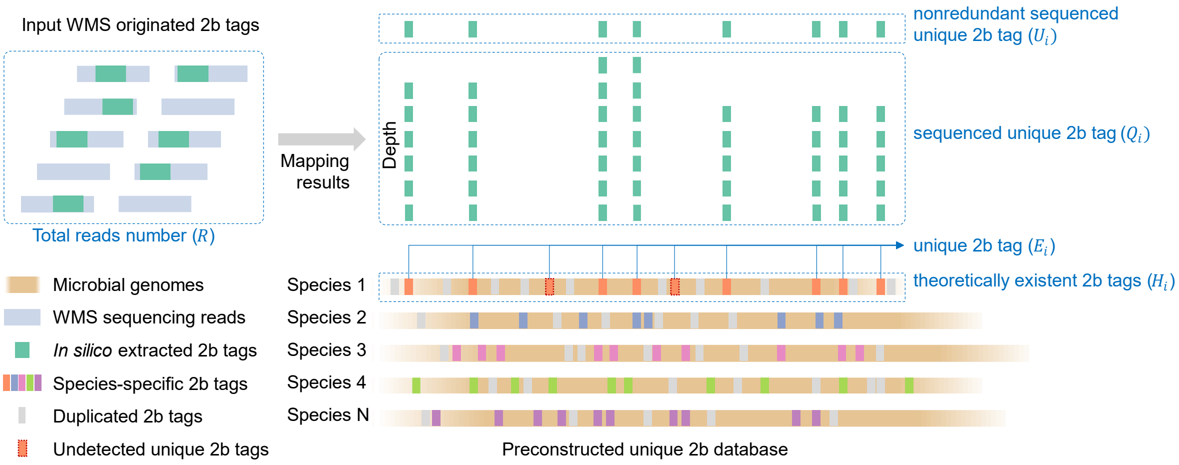

Figure S7. Definition of the terminology used in the species identification procedures of MAP2B. For species-i, we denote its total number of 2b tags generated by *in silico* digestion of its genome as H_i. Among the H_i tags, there are E_i tags that are single-copy within the genome of species-i, and are unique to species-i w.r.t all other species in the database of microbial genomes. Given an input WMS sequencing dataset, we *in silico* extract 2b tags, map them to the species-specific 2b tags, and denote the number of tags unique to species-i as Q_i. Among the Q_i tags unique to species-i, there are U_i distinct or nonredundant ones. R is the total number of reads in the WMS sequencing data, which can vary a lot across different samples.

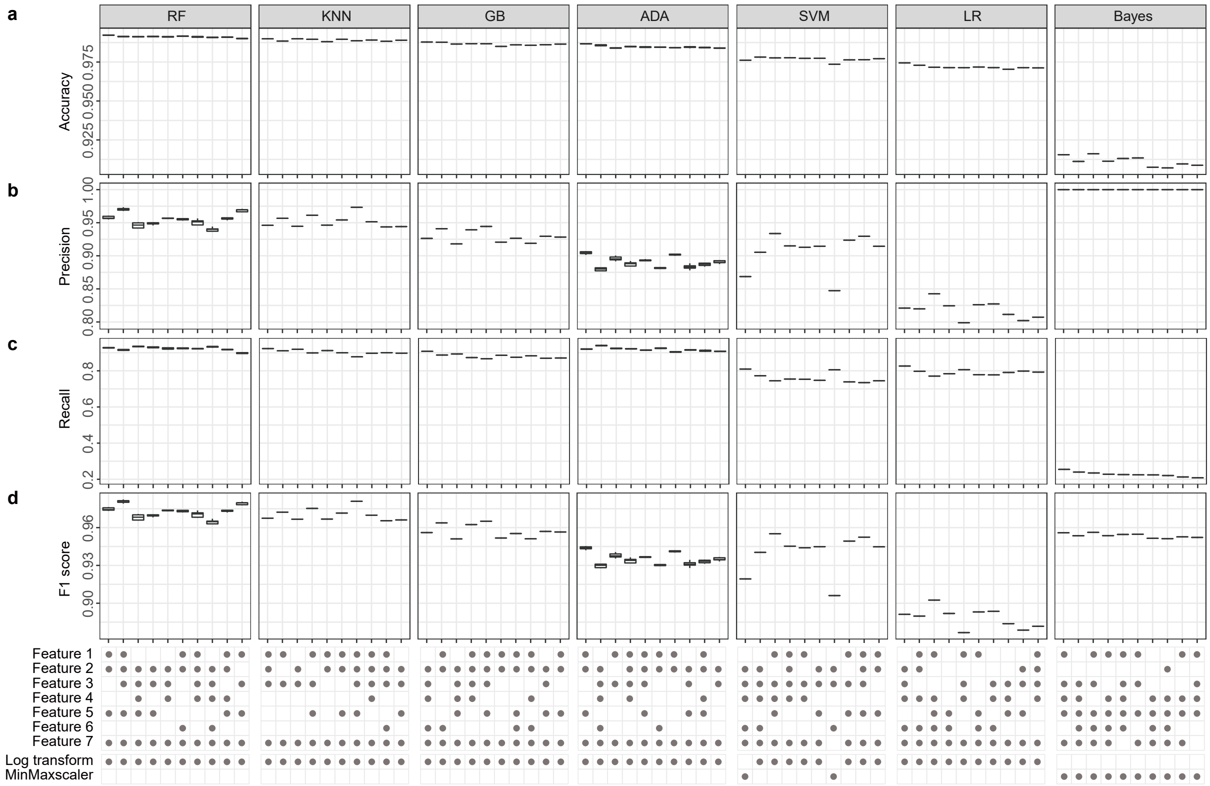

Figure S8. TOP-10 feature combinations for each classifier in discriminating false positives from true positives. To select the best feature combination, and the best classifier, both min-max scaling and log transformation were used separately for any combination of the seven features before passing to seven classifiers: Random Forest (RF), K-neighbors (KNN), Gradient Boosting (GB), AdaBoost (ADA), Support-Vector Machines (SVM), Logistic Regression (LR), and naive Bayes (Bayes). For each feature combination and each classifier, we performed five-fold cross-validation five times using the CAMI2 simulation datasets and compared their performance via (**a**) Accuracy, (**b**) Precision, (**c**) Recall, and (**d**) F1 score.

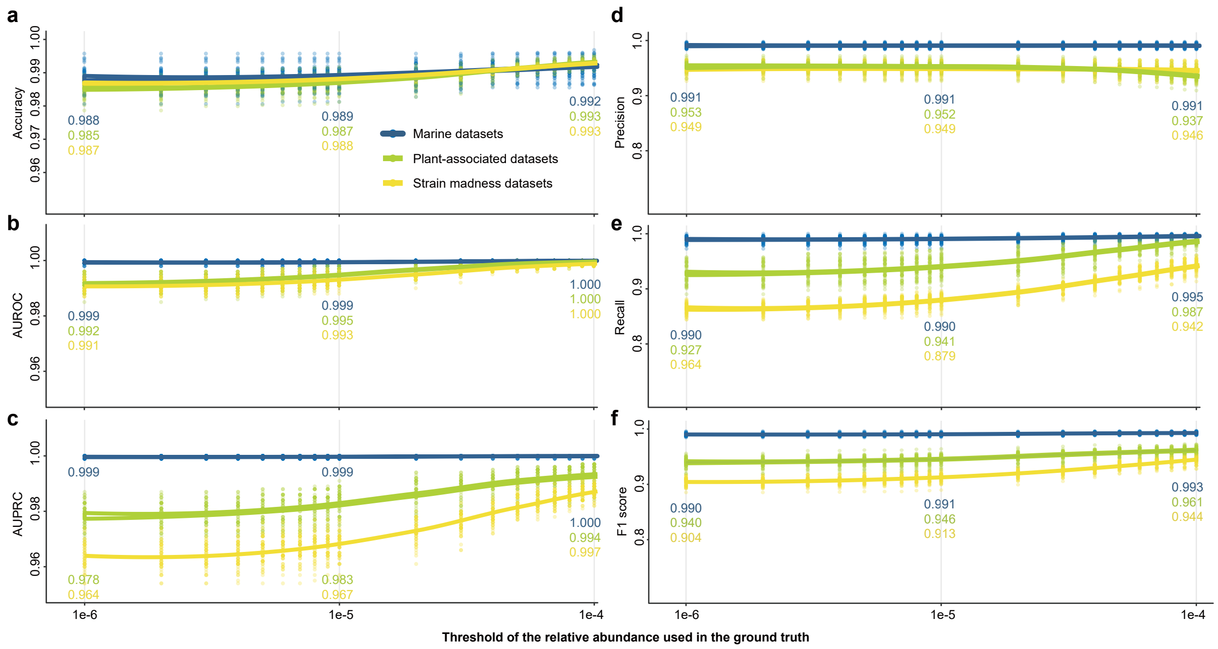

Figure S9. Performance evaluations of the machine learning model on false positive recognition based on 50 times five-fold cross-validation. When evaluating the performance of the model, the low-abundance species in the ground truth were filtered out according to different abundance thresholds. We gradually discard the true species with relative abundance from 10^{-6} to 10^{-4} and illustrate the performance of the model in determining false positives using metrics such as (**a**) the Area Under the Receiver Operating Characteristic (AUROC) curve; (**b**) the Area Under the Precision-Recall Curve (AUPRC); (**c**) Accuracy; (**d**) Precision; (**e**) Recall; and (**f**) F1-score.

Reference

1. Parks, D.H. et al. A complete domain-to-species taxonomy for Bacteria and Archaea. *Nat Biotechnol* **38**, 1079-1086 (2020).
2. Yates, A.D. et al. Ensembl Genomes 2022: an expanding genome resource for non-vertebrates. *Nucleic Acids Res* **50**, D996-D1003 (2022).
3. Meyer, F. et al. Critical Assessment of Metagenome Interpretation: the second round of challenges. *Nature methods* **19**, 429-+ (2022).
4. Wood, D.E., Lu, J. & Langmead, B. Improved metagenomic analysis with Kraken 2. *Genome biology* **20**, 257 (2019).
5. Lu, J., Breitwieser, F.P., Thielen, P. & Salzberg, S.L. Bracken: estimating species abundance in metagenomics data. *PeerJ Computer Science* **3**, e104 (2017).
6. Blanco-Miguez, A. et al. Extending and improving metagenomic taxonomic profiling with uncharacterized species using MetaPhlAn 4. *Nat Biotechnol* (2023).
7. Ruscheweyh, H.J. et al. Cultivation-independent genomes greatly expand taxonomic-profiling capabilities of mOTUs across various environments. *Microbiome* **10**, 212 (2022).
8. Breitwieser, F.P., Baker, D.N. & Salzberg, S.L. KrakenUniq: confident and fast metagenomics classification using unique k-mer counts. *Genome biology* **19**, 198 (2018).
