# Supplementary material for: Removal of false positives in metagenomics-based taxonomy profiling via targeting Type IIB restriction sites
Source: Nat Commun. 2023 Sep 1;14:5321. doi: 10.1038/s41467-023-41099-8 (PMC10474111; doi:10.1038/s41467-023-41099-8)
Supplement: Supplementary file 3 — Reporting Summary [file 41467_2023_41099_MOESM3_ESM.pdf]

nature portfolio

Corresponding author(s): Yang-Yu Liu

Last updated by author(s): Aug 19, 2023

Reporting Summary

Nature Portfolio wishes to improve the reproducibility of the work that we publish. This form provides structure for consistency and transparency in reporting. For further information on Nature Portfolio policies, see our Editorial Policies and the Editorial Policy Checklist.

Statistics

For all statistical analyses, confirm that the following items are present in the figure legend, table legend, main text, or Methods section.

n/a	Confirmed	
[ ]	[x]	The exact sample size (*n*) for each experimental group/condition, given as a discrete number and unit of measurement
[ ]	[x]	A statement on whether measurements were taken from distinct samples or whether the same sample was measured repeatedly
[ ]	[x]	The statistical test(s) used AND whether they are one- or two-sided<br>*Only common tests should be described solely by name; describe more complex techniques in the Methods section.*
[x]	[ ]	A description of all covariates tested
[x]	[ ]	A description of any assumptions or corrections, such as tests of normality and adjustment for multiple comparisons
[ ]	[x]	A full description of the statistical parameters including central tendency (e.g. means) or other basic estimates (e.g. regression coefficient) AND variation (e.g. standard deviation) or associated estimates of uncertainty (e.g. confidence intervals)
[ ]	[x]	For null hypothesis testing, the test statistic (e.g. *F*, *t*, *r*) with confidence intervals, effect sizes, degrees of freedom and *P* value noted<br>*Give P values as exact values whenever suitable.*
[x]	[ ]	For Bayesian analysis, information on the choice of priors and Markov chain Monte Carlo settings
[x]	[ ]	For hierarchical and complex designs, identification of the appropriate level for tests and full reporting of outcomes
[x]	[ ]	Estimates of effect sizes (e.g. Cohen's *d*, Pearson's *r*), indicating how they were calculated

Our web collection on statistics for biologists contains articles on many of the points above.

Software and code

Policy information about availability of computer code

Data collection	Simulated WMS data in this paper were: (1) downloaded from CAMI2 (https://www.nature.com/articles/s41592-022-01431-4#data-availability, PUBLISSO with DOIs: https://doi.org/10.4126/FRL01-006425521); (2) generated by self-programmed R scripts ,which can be found here: https://github.com/sunzhengCDNM/MAP2B/tree/master/Manuscript/Figure3/WMS%20simulation.<br>As for real sequencing data of: (1) ATCC mock community MSA 1002, it can be downloaded at: https://doi.org/10.6084/m9.figshare.21627077.v3 or at NCBI with the project number PRJNA1006621; (2) the publicly available dataset used in this paper, the metagenomic sequences for the PRISM, LifeLines DEEP and NLIBD cohorts are available via SRA with BioProject number PRJNA400072. Metabolomics data (accession number PR000677) are available at the National Institutes of Health Common Fund's Metabolomics Data Repository and Coordinating Center.
Data analysis	For taxonomic profiling of the simulated metagenomic shotgun sequencing data, we used MetaPhlAn3 (version 3.0.2; https://github.com/biobakery/MetaPhlAn), mOTUs2 (version 2.5.1; https://github.com/motu-tool/mOTUs_v2), Kraken2 (version 2.1.1; https://github.com/DerrickWood/kraken2), Bracken (version 2.5.0; https://github.com/jenniferlu717/Bracken), KrakenUniq (version 0.5.8; https://github.com/fbreitwieser/krakenuniq), MAP2B (version 1.3; https://github.com/sunzhengCDNM/MAP2B).<br>For prediction of metabolic profiles,the pipelines can be found at https://github.com/YDaiLab/MiMeNet and https://github.com/wt1005203/mNODE.<br>For beta diversity analysis, calculation of AUROC/AUPRC, and data visualization, the R code can be found in https://github.com/sunzhengCDNM/MAP2B/tree/master/Manuscript.

For manuscripts utilizing custom algorithms or software that are central to the research but not yet described in published literature, software must be made available to editors and reviewers. We strongly encourage code deposition in a community repository (e.g. GitHub). See the Nature Portfolio guidelines for submitting code & software for further information.

Data

Policy information about availability of data

All manuscripts must include a data availability statement. This statement should provide the following information, where applicable:

- Accession codes, unique identifiers, or web links for publicly available datasets
- A description of any restrictions on data availability
- For clinical datasets or third party data, please ensure that the statement adheres to our policy

We've submitted the WMS data of the ATCC MOCK MSA 1002 to NCBI with the project number PRJNA1006621, which can also be accessed via figshare and can be downloaded at: https://doi.org/10.6084/m9.figshare.21627077.v3.
As for all simulation data (e.g., in Fig. 3, Fig S3, Fig S4), users can download the scripts and reproduce them to avoid the heavy download task. The scripts are available in the "Manuscript/Figure3/WMS simulation" folder on our GitHub repository.

Research involving human participants, their data, or biological material

Policy information about studies with human participants or human data. See also policy information about sex, gender (identity/presentation), and sexual orientation and race, ethnicity and racism.

Reporting on sex and gender	NA.
Reporting on race, ethnicity, or other socially relevant groupings	NA.
Population characteristics	NA.
Recruitment	NA.
Ethics oversight	NA.

Note that full information on the approval of the study protocol must also be provided in the manuscript.

Field-specific reporting

Please select the one below that is the best fit for your research. If you are not sure, read the appropriate sections before making your selection.

☒ Life sciences ☐ Behavioural & social sciences ☐ Ecological, evolutionary & environmental sciences

For a reference copy of the document with all sections, see nature.com/documents/nr-reporting-summary-flat.pdf

Life sciences study design

All studies must disclose on these points even when the disclosure is negative.

Sample size	Our sample size references benchmark works like the CAMI2 simulation datasets (minimum 10 for different scenarios).<br>130 simulation WMS data from CAMI2 (marine, strain-madness, plant-associated) were used to train the false positive recognition model and for the five-fold cross-validation. Please see Figure S9.<br>We simulated 54 (using random microbial genomes in the NCBI ResSeq) + 30 (using intersection of different metagenomic profilers' databases) WMS data + 54 (using random microbial genomes in the NCBI ResSeq with 1-3% mutation rate) to illustrate the differential benchmarking results of four representative metagenomics profilers. Simulation for WMS data considered the sequencing depth and species richness in various situations (e.g., 10-500 species, 7.5 million reads to 150 million reads, 1-3% mutation rate, 1000-10000 independent microbial genomes). Please see Figure 3, Figure S3 and Figure S4.<br>All the individuals (n=220, n=155 for training, and n=65 for testing) from the PRISM, LifeLines DEEP, and NLIBD cohorts (publicly available) with metagenomic sequencing and metabolomics data were used to test the accuracy of predicting metabolomics profile using taxonomic profiles generated by different metagenomic profilers.
Data exclusions	No data were excluded from the analyses.
Replication	In the cross validation of the false positive recognition model, the 5 fold cross validation were repeated 50 time and the mean value were used to avoid bias.
Randomization	The main focus of this paper is benchmarking the output results of different software. This means comparing the output results of all software with the ground truth and further comparing the quantified differences. Therefore, there is no grouping of control and experimental groups。<br>In WMS sequencing reads and profile simulation, the microbial species/genomes were selected randomly.
Blinding	To validate and compare the performance of MAP2B, we generated metagenomic sequencing data with known taxonomic profiles. We used the default parameters for all metagenomic profilers in this study. This means that the output results for any specific metagenomic sequencing data are almost fixed. Therefore, whether or not the blinding is involved, it does not affect the benchmarking results.

Reporting for specific materials, systems and methods

We require information from authors about some types of materials, experimental systems and methods used in many studies. Here, indicate whether each material, system or method listed is relevant to your study. If you are not sure if a list item applies to your research, read the appropriate section before selecting a response.

Materials & experimental systems

n/a	Involved in the study	
☒	☐	Antibodies
☒	☐	Eukaryotic cell lines
☒	☐	Palaeontology and archaeology
☒	☐	Animals and other organisms
☒	☐	Clinical data
☒	☐	Dual use research of concern
☒	☐	Plants

Methods

n/a	Involved in the study	
☒	☐	ChIP-seq
☒	☐	Flow cytometry
☒	☐	MRI-based neuroimaging
